# Hepatic Epithelioid Angiosarcoma with Abberrant Synaptophysin and CDX2 Expression Mimicking Neuroendocrine/Metastatic Carcinoma: A Fatal Case Report

**DOI:** 10.14309/crj.0000000000002389

**Published:** 2026-11-04

**Authors:** Muhammad Usman, Muhammad Usman Shahbaz, Arsalan Ahmed, Muahammad Zawar Asif, Adnan Memon, Laiba Murtaza, Rafif Ahmed

**Affiliations:** 1 Mercy Hospital, Fort Smith, AR; 2 Arkansas College of Osteopathic Medicine, Fort Smith, AR; 3 Liaquat University of Medical and Health Sciences, Jamshoro, Pakistan; 4 King Edward Medical University, Lahore, Pakistan; 5 Allama Iqbal Medical College, Lahore, Pakistan

**Keywords:** primary hepatic angiosarcoma, epithelioid angiosarcoma, hyponatremia, case report, hepatic malignancy

## Abstract

We report a fatal case of presumed primary hepatic epithelioid angiosarcoma (PHEA) in an 82-year-old woman with back pain, abdominal distension, and hyponatremia. Imaging showed ascites and hypodense hepatic lesions. Ascitic cytology was negative. Liver biopsy showed malignant cells with positive erythroblast transformation-specific regulated gene 1 (ERG) and cluster of differentiation 31 (CD31) on immunohistochemistry. Patchy synaptophysin and caudal-type homeobox 2 positivity initially suggested a neuroendocrine tumor but proved aberrant; negative chromogranin with ERG/CD31 positivity supported PHEA. She was transitioned to hospice care due to rapid clinical decline. This case highlights the atypical presentation, diagnostic pitfalls, and aggressive clinical course of PHEA.

## INTRODUCTION

Primary hepatic angiosarcoma is a rare and aggressive vascular tumor, accounting for 1%–2% of primary malignancies of the liver.^1,2^ While usually idiopathic, exposure to carcinogens like thorium dioxide, arsenic, and anabolic steroids is linked with disease development.^2,3^

The epithelioid subtype is aggressive with distinct epithelioid morphology, leading to diagnostic challenges.^2,4,5^

Computed tomography (CT) or magnetic resonance imaging typically shows multifocal, heterogeneous, or infiltrative hepatic lesions, though these mimic other malignancies.^2,6^ The definitive diagnosis requires histopathological evaluation and immunohistochemistry (IHC), with endothelial markers such as cluster of differentiation 31 (CD31), cluster of differentiation 34, and erythroblast transformation-specific related gene 1 (ERG) being highly sensitive and specific.^1,4^

Primary hepatic epithelioid angiosarcoma (PHEA) has a dismal prognosis due to rapid progression, hemorrhage, or hepatic failure.^2^ We present a case of PHEA in an elderly woman with atypical presentation and a rapidly fatal clinical course.

## CASE REPORT

An 82-year-old woman with a history of generalized anxiety disorder and chronic lower back pain presented with 2 weeks of worsening lower back pain, bilateral lower extremity weakness, and abdominal distension. The patient denied smoking, alcohol use, past chemical exposure, or saddle anesthesia. She also reported 6 months of unintentional weight loss.

Physical examination was significant for abdominal distension with mild diffuse tenderness without guarding or rebound tenderness and 2+ bilateral lower extremity edema.

Initial laboratory test results revealed microcytic anemia (Hb 6.8 g/dL [11.2–15.7], mean corpuscular volume 59.0 fL [79.0–92.2]), leukocytosis (12.4 K/uL [4.0–10.0]), reactive thrombocytosis (514 K/uL [163–337]), and severe hyponatremia (112 mmol/L [136–145]). Hypokalemia (3.2 mmol/L [3.5–5.1]) and hypoalbuminemia (2.4 g/dL [4.0–4.9]) were present. Liver enzymes were mildly elevated with normal prothrombin time/international normalized ratio panels. Iron studies and serum ferritin were consistent with multifactorial anemia.

Abdominal and pelvic contrast-enhanced CT revealed large-volume ascites with bilateral pleural effusions; multiple heterogeneous hypodense liver lesions, the largest measuring 4.6 cm; and a 6.1 cm complex left ovarian cyst, suspicious for malignancy (Figure 1). Lumbar spinal magnetic resonance imaging was negative for any metastasis.

**Figure 1.**
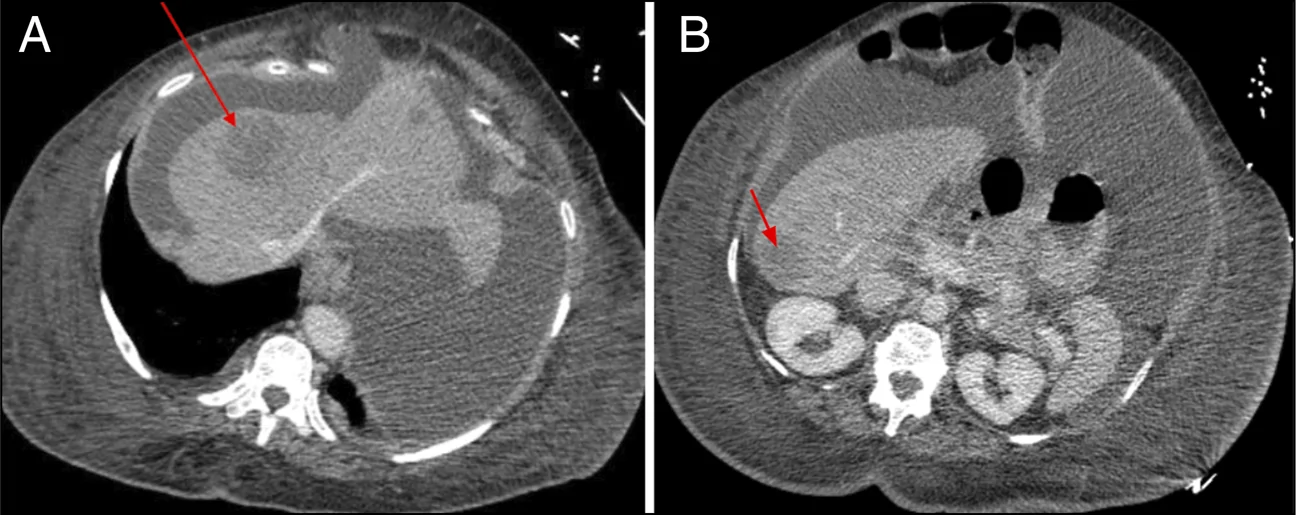
Contrast-enhanced axial CT of the abdomen. (A) Superior axial image showing a heterogeneous hypodense lesion in the right hepatic lobe (arrow) with large-volume ascites and a right basal pleural effusion. (B) A more caudal axial image demonstrating an additional peripheral heterogeneous hypodense hepatic lesion (arrow), with extensive perihepatic ascites. CT, computed tomography.

The patient developed rapid abdominal distension and hypoxia secondary to effusions, requiring oxygen support. She was started on spontaneous bacterial peritonitis prophylaxis, and 8 L of nonbloody, straw-colored, ascitic fluid was removed by paracentesis. Serum-ascites albumin gradient was >1.1, total protein 3.5 g/dL, albumin 1.7 g/dL, white blood cell count 154/uL with 13% neutrophils, red blood cell count <2000/uL, and lactate dehydrogenase 607 U/L; cultures were negative; cell block-based cytology with immunostaining was negative; and tumor markers (carbohydrate antigen 19-9, alpha-fetoprotein, and cancer antigen 125) were normal.

Owing to rapid reaccumulation of ascitic fluid, repeat paracentesis (6.3 L) again showed negative cytology. The patient required hypertonic saline for hypervolemic hypo-osmolar hyponatremia (osmolality: 238 mOsm/kg). Hemoglobin after 1 unit blood transfusion and coagulation parameters remained stable throughout.

Eventually, an ultrasound-guided liver biopsy revealed a poorly differentiated high-grade malignant tumor with neoplastic cells infiltrating sinusoidal spaces. IHC was positive for ERG and CD31 with a Ki-67 index >90%; patchy positive for synaptophysin and caudal-type homeobox 2 (CDX2); and negative for cluster of differentiation 117, desmin, chromogranin, and chymotrypsin (Figure 2). Negative calmodulin-binding transcription activator 1 staining ruled out epithelioid hemangioendothelioma, leading to the diagnosis of epithelioid angiosarcoma.

**Figure 2.**
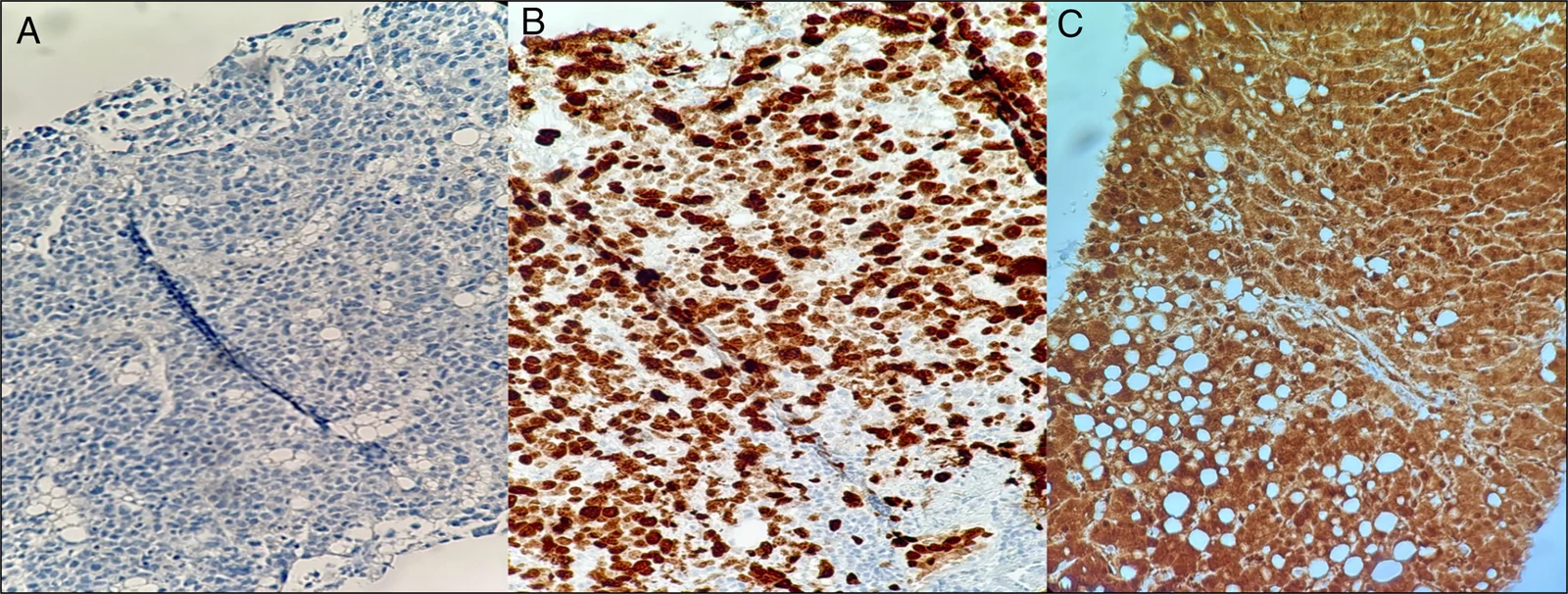
(A) Hematoxylin and eosin (H&E) staining (original magnification ×10) showing sheets of atypical epithelioid cells with poorly formed vascular channels. (B) Erythroblast transformation-specific related gene immunostaining showing diffuse strong nuclear positivity (original magnification ×10). (C) Cluster of differentiation 31 (CD31) immunostaining showing strong cytoplasmic/membranous positivity (original magnification ×10).

Despite aggressive management, the patient continued to have recurrent ascites and hyponatremia requiring hypertonic saline repeatedly in the intensive care unit. Given her advanced malignancy, poor prognosis, advanced age, and marginal performance status, she was a poor candidate for chemotherapy or immunotherapy. Following a multidisciplinary goals-of-care discussion, the patient transitioned to hospice and died on day 12 from progressive hepatic failure.

## DISCUSSION

Hepatic angiosarcoma itself accounts for 1%–2% of primary liver malignancies, which characteristically affects older men^1–3^; its occurrence as the aggressive epithelioid subtype in an 82-year-old woman is rarely documented in the literature.^4^

Clinical presentation is often nonspecific, including fatigue, abdominal pain, distension, anemia, coagulopathy, and paraneoplastic syndromes, and leads to diagnostic delay.^2,5^ In our case, atypical manifestations like hyponatremia from massive ascites-related hypervolemia; mechanical back pain related to ascites and deconditioning with metastasis-negative spinal magnetic resonance imaging; multifactorial anemia; and absence of coagulopathy contributed to diagnostic delay and highlight the diverse nature of disease presentation.^8^

Negative cytology, despite extensive hepatic infiltration in our case, reflects the tendency of angiosarcoma cells to remain cohesive within sinusoidal/vascular channels and exfoliate poorly, so a negative paracentesis does not exclude vascular malignancy.^4^

Radiological findings in HA are variable and nonspecific, often featuring multifocal, infiltrative, hemorrhagic, or necrotic lesions that mimic other malignancies.^6,9^ In our case, multifocal hepatic burden with no extrahepatic primary on abdominal/pelvic computed tomography and spinal magnetic resonance imaging, normal tumor markers, and negative ascitic cytology supported primary hepatic origin.^6,9^ The key teaching point in our case is that patchy synaptophysin and CDX2 expression can mimic a neuroendocrine tumor or metastatic carcinoma. These were interpreted as aberrant expression, since chromogranin was negative and diffuse endothelial marker positivity with epithelioid nonvasoformative morphology is diagnostic of epithelioid angiosarcoma.^4^

The incidental ovarian cyst was considered less likely a source due to normal cancer antigen 125 and an endothelial (ERG+/CD31+) IHC.^1,4^ Our patient's strong positivity for CD31 and ERG, and negative calmodulin-binding transcription activator 1, confirmed the diagnosis. Since positron emission tomography scan and autopsy were not performed, given rapid deterioration and the patient's wishes, “primary” is used as a clinicopathologic designation in our case. This workup including radiography, histopathology, and IHC excluded other differentials including hepatocellular carcinoma, ovarian cancer, lymphoma, and neuroendocrine tumors.

Management is stage dependent: Surgical resection is only viable for localized disease.^5,10^ For advanced disease, anthracycline and taxane-based systemic therapies are principal options, with antiangiogenic and immunotherapeutic approaches investigational and outcomes generally poor.^2,7,10^ In our case, various patient factors and extensive disease burden precluded treatment and a palliative approach was adopted.

PHEA has a dismal prognosis; the median survival may be as short as approximately 114 days.^11^ The epithelioid subtype and primary hepatic origin are independent adverse prognostic factors with mortality usually resulting from hemorrhage or hepatic failure.^2,5,7^ Our patient's rapid decline and death within days reflects this aggressive pattern.

This case underscores important clinical lessons, including the diagnostic limitations of imaging, crucial role of histopathology and IHC in confirming the diagnosis particularly when aberrant neuroendocrine and epithelial marker expression can masquerade other malignancies, and potential for atypical presentations.

## DISCLOSURES

Author contributions: M. Usman identified the case, performed the literature review, acquired the images, and drafted the manuscript. MU Shahbaz contributed to study conceptualization, data collection, literature review, and manuscript editing. A. Ahmed assisted with literature review, manuscript formatting, and reference management. MZ Asif and L. Murtaza critically revised the manuscript and contributed to clinical interpretation. A. Memon and R. Ahmed contributed to data collection, manuscript editing, and proofreading. All authors approved the final manuscript. M. Usman is the article guarantor.

Financial disclosure: None to report.

Previous presentation: Poster presentation at Digestive Disease Week, May 2-5, 2026; Chicago, IL.

Informed consent was obtained for this case report.

## ABBREVIATIONS:

CD31: cluster of differentiation 31
CDX2: caudal-type homeobox 2
CT: computed tomography
ERG: erythroblast transformation-specific related gene 1
H&E: hematoxylin and eosin
IHC: immunohistochemistry
MRI: magnetic resonance imaging
PHEA: primary hepatic epithelioid angiosarcoma
